# Effect of Iron Deficiency on c-kit^+^ Cardiac Stem Cells In Vitro

**DOI:** 10.1371/journal.pone.0065721

**Published:** 2013-06-10

**Authors:** Dongqiang Song, Yuanmin Li, Jiatian Cao, Zhihua Han, Lin Gao, Zuojun Xu, Zhaofang Yin, Guifang Wang, Yuqi Fan, Changqian Wang

**Affiliations:** 1 Department of Cardiology, Ninth People’s Hospital, Shanghai Jiaotong University Medical School, Shanghai, PR China; 2 Shanghai Key Laboratory of Stomatology, Ninth People’s Hospital, Shanghai Jiaotong University Medical School, Shanghai, PR China; Tokai University, Japan

## Abstract

**Aim:**

Iron deficiency is a common comorbidity in chronic heart failure (CHF) which may exacerbate CHF. The c-kit^+^ cardiac stem cells (CSCs) play a vital role in cardiac function repair. However, much is unknown regarding the role of iron deficiency in regulating c-kit^+^ CSCs function. In this study, we investigated whether iron deficiency regulates c-kit^+^ CSCs proliferation, migration, apoptosis, and differentiation in vitro.

**Method:**

All c-kit^+^ CSCs were isolated from adult C57BL/6 mice. The c-kit^+^ CSCs were cultured with deferoxamine (DFO, an iron chelator), mimosine (MIM, another iron chelator), or a complex of DFO and iron (Fe(III)), respectively. Cell migration was assayed using a 48-well chamber system. Proliferation, cell cycle, and apoptosis of c-kit^+^ CSCs were analyzed with BrdU labeling, population doubling time assay, CCK-8 assay, and flow cytometry. Caspase-3 protein level and activity were examined with Western blotting and spectrophotometric detection. The changes in the expression of cardiac-specific proteins (GATA-4,TNI, and β-MHC) and cell cycle-related proteins (cyclin D1, RB, and pRB) were detected with Western blotting.

**Result:**

DFO and MIM suppressed c-kit^+^ CSCs proliferation and differentiation. They also modulated cell cycle and cardiac-specific protein expression. Iron chelators down-regulated the expression and phosphorylation of cell cycle-related proteins. Iron reversed those suppressive effects of DFO. DFO and MIM didn’t affect c-kit^+^ CSCs migration and apoptosis.

**Conclusion:**

Iron deficiency suppressed proliferation and differentiation of c-kit^+^ CSCs. This may partly explain how iron deficiency affects CHF prognosis.

## Introduction

Cardiac stem cells (CSCs) are the resident cardiac stem cells which were first found in adult heart in 2003. CSCs can proliferate and differentiate into cardiomyocytes, vascular smooth muscle cells, and endothelial cells in vitro and vivo [1]. In vivo, cardiomyocytes renewal by CSCs occurs every year [2]. CSCs can migrate to the injured myocardium to reconstitute the myocardium and improve cardiac function after Ischemia/reperfusion injury [3]. CSCs can be grouped by surface markers such as c-kit, Sca-1, MDR1, and Isl-1 [4]. The ability of c-kit^+^ CSCs to differentiate into cardiomyocytes is 2.3-, 5.9-, and 7.6-fold greater than MDR1^+^, Sca-1^+^, and c-kit-MDR1-Sca-1^+^ CSCs, respectively [5]. Thus, c-kit^+^ CSCs are the most frequently investigated cardiac stem cells.

Chronic heart failure (CHF), one of the primary causes of death in the world [6], is commonly accompanied by iron deficiency [7]. Recent researches have revealed that there are many causes of iron deficiency in CHF [8], and that iron deficiency exacerbates CHF [9]. CHF patients with iron deficiency experience worse symptoms, have less exercise capacity, undergo poorer prognosis than those without. Iron replacement therapy improves CHF patients’ symptoms, exercise capacity, and quality of life [10]. However, much is still not clear regarding the specific role of iron deficiency in CHF.

Considering the important roles of c-kit^+^ CSCs and iron in cardiac function, we speculated that iron deficiency may modulate the function of c-kit^+^ CSCs, resulting in worse prognosis in CHF patients. To demonstrate this postulation, we investigated the effect of iron deficiency on c-kit^+^ CSCs proliferation, migration, apoptosis, and differentiation in this study.

## Materials and Methods

All experimental protocols involving cells or procedures with mice were approved by the Ethics Committee of Shanghai Jiao Tong University School of Medicine.

### Isolation of c-kit^+^ CSCs

All c-kit^+^ CSCs were isolated as a previous report with minor modifications [11]. Briefly, hearts were removed under aseptic conditions from adult C57BL/6 mice. The myocardial tissue was cut into 1 to 2 mm^3^ pieces, washed twice with Ca^2+^–Mg^2+^–free phosphate-buffered solution (PBS) and digested four times for 10 min with 0.05% collagenase II (Invitrogen, Carsbad, CA) alternately at 37°C with frequent shaking. After the enzymatic digestion, a cell suspension was collected and filtered with a strainer. The obtained cells were incubated with lineage cell depletion kit (Miltenyi biotec, Germany) and separated by biotin-antibody cocktail immunomagnetic microbeads (Miltenyi biotec, Germany). The negative lineage cells were collected and incubated with anti-mouse-c-kit microbeads (Miltenyi biotec, Germany) and separated. Those c-kit positive small round cells were collected.

### Immunostaining

Immunostaining was performed by incubation with specific primary antibody: rabbit anti-c-kit (diluted 1∶100, Santa Cruz Biotechnology, USA) at 4°C overnight. Cells were then incubated with FITC-conjugated rat anti-rabbit Ig G (diluted 1∶250) at 25°C for 2 h. Nuclei were stained with DAPI (Sigma, USA).The immunoreactions were observed under a fluorescent microscope (Nikon, Japan).

### Cell Culture and Flow Cytometric Analysis

The obtained c-kit^+^ CSCs were cultured with DMEM (Thermo, USA) containing 15% fetal calf serum (FCS), 10 ng/ml bFGF, and 10 ng/ml LIF at 37°C in a humidified atmosphere with 5% CO_2_ for three days. After recovery, the cells were used for flow cytometric analysis and subsequent experiments.

Then, the expression of cell surface marker of the collected cells was analyzed. The collected cells were made into single cells suspensions and stained with FITC-conjugated antibodies against c-kit, CD34, CD45 (Invitrogen, Carlsbad, CA), or IgG isotype control (Cell signaling, Massachusetts, USA). The positive rate of c-kit, CD34, CD45, or isotype control in the total cells was detected with flow cytometry.

### Quantification of Intracellular Iron Content

Cells were treated with DFO (Sigma, USA) (0, 50, 100, 200 µM), MIM (Sigma, USA) (0.5 mM), or a complex of DFO (100 µM) and Fe(III) (0.5 mM). Intracellular iron content was determined by the colorimetric ferrozine-based assay as previously described [12]. Absorbance of each sample was measured at 562 nm using the Spectra Max microplate reader.

### Cell Proliferation and BrdU Incorporation Analysis

Isolated c-kit^+^ CSCs were seeded on 96-well plates (5×10^3^ cells per well), and cultured in 100 µl 10% FCS DMEM medium for 12 h. Cells were treated with different concentrations of DFO (0, 50, 100, 200 µM) or MIM (0.5 mM) for different time (24, 48, 72 h). The cellular level was measured with Cell Counting Kit-8 (CCK-8) (Dojindo, Japan) and a Microplate Reader (450 nm). For BrdU (Sigma) incorporation analysis,cells were treated with DFO (100 µM) for 48 h. After labeling with BrdU for the final 18 h of the incubation period, DNA was denatured and cells were incubated with anti-BrdU monoclonal antibody for detecting incorporated BrdU. The absorbance related to the BrdU level was measured with a microplate reader at 450 nm. Then, the proliferation index for each group was calculated in relation to the corresponding control taken to be 100%.

### Population Doubling Time

A total of 2×10^5^ cells/ml of c-Kit^+^ CSCs at passage 3 was cultivated for a period of 3 days (24, 48 and 72 h). Cells were treated with DFO (100 µM), MIM (0.5 mM), or a complex of DFO (100 µM) and Fe(III) (0.5 mM). Cell numbers were determined every 24 h using a Neubauer improved haematocytometer (Sigma-Aldrich, USA). Cell numbers were counted in triplicate. Population doubling time was calculated using online algorithm software provided at http://www.doubling-time.com.

### Cell Cycle Analysis

Flow cytometry analysis of cell cycle was performed as a previous report [13]. Briefly, c-Kit^+^ CSCs were treated with/without DFO, MIM, or a complex of DFO and Fe(III). After treatment, cells were harvested, trypsinized, and fixed in 70% ethanol overnight at −20°C. Cells were washed with PBS, treated with RNase for 30 min, followed by staining with propidium iodide. The DNA content was analyzed with FACSCalibur and CellQuest Pro software. Results were obtained from 5 independent experiments.

### Cell Migration Assay

The c-Kit^+^ CSCs were pre-incubated with DFO (100 µM), MIM (0.5 mM), or a complex of DFO (100 µM) and Fe(III) (0.5 mM) for 24 h before seeding. Then, 1×10^4^ c-Kit^+^ CSCs in 100 µl of medium were seeded into the upper chamber (pore size, 8 µm). DMEM plus 10% FCS was put into the lower chamber of a 24-well transwell. The chamber was incubated for 12 h at 37°C in a humidified atmosphere with 5% CO_2_ before the filter and the un-migrated cells were removed. After fixation and staining, the number of migrated cells on the lower surface of the filter was counted under a microscope. Experiments were repeated 5 times.

### Cell Apoptosis Assay

The c-Kit^+^ CSCs were stimulated with DFO (100 µM), MIM (0.5 mM), H_2_O_2_ (0.5 mM), or a complex of DFO (100 µM) and Fe(III) (0.5 mM) for 24 h or 48 h. The apoptosis of c-Kit^+^ CSCs were then analyzed by flow cytometry with Annexin V and PI staining (Invitrogen, Carsbad, CA). Cells were harvested and washed in cold phosphate-buffered saline (PBS) before they were re-suspended in 1X Binding Buffer at a concentration of 1×10^6^ cells/ml. Then, 100 µl of cell suspension were treated with 5 µl of FITC Annexin V and 1 µl 100 µg/ml propidium iodide (PI). The cells were incubated for 15 min at room temperature (25°C), and 400 µl of 1X Binding Buffer was added to each tube. The stained cells were analyzed by flow cytometer as soon as possible.

### Quantification of Caspase-3 Activity

The activity of caspase-3 was assessed using Caspase-3 Colorimetric Assay Kits (Beyotime, China). In brief, cells were incubated with DFO (100 µM), MIM (0.5 mM), H_2_O_2_ (0.5mM), or a complex of DFO (100 µM) and Fe(III) (0.5 mM), for 24 h or 48 h. The cells were then washed in PBS and suspended in 100 ml lysis buffer (20 mM HEPES, pH 7.9, 20% glycerol, 200 mM KCl, 0.5 mM EDTA, 0.5% NP-40, 0.5 mM DTT, 1% protease inhibitor cocktail) for 30 min on ice. The cell lysates were centrifuged at 12, 000 g at 4°C for 20 min and then collected. Protein concentration was determined by the Bradford method. Supernatant samples containing 50 mg of total proteins were used for determination of caspase-3 activity. These samples were added to each well in 96-well microtiter plates with the DEVD-pNA at 37°C for 2 h. The optical density of each well was measured at 405 nm using a microplate reader.

### Cell Differentiation Assay

The c-Kit^+^ CSCs were plated on 6-well plates. Cell differentiation was induced by differentiation medium (DMEM, 10% FCS and 10^−8^ M dexamethasone) with/without DFO, MIM, or a complex of DFO and Fe(III) for 2 weeks. After the induction of differentiation, total proteins were extracted. The ability of differentiation was determined by analyzing the cellular morphology and the expression of cardiac-specific markers (TNI, β-MHC, and GATA-4).

### Preparation of Cytoplasmic and Nuclear Extracts

Cells were rinsed in cold PBS and lysed in a solution containing 0.6% Nonidet P-40, 10 mM KCl, 10 mM HEPES, 0.1 mM EDTA (all products from Sigma Aldrich), and Complete™ Mini-EDTA-free protease inhibitor cocktail (Roche Diagnostics). After centrifugation (30 sec, 2,000×g), supernatants were incubated on ice for 5 min. Nuclei were precipitated by centrifugation (4°C, 3 min, 15,000×g), supernatants collected as cytosolic extracts, and the nuclei re-suspended in a solution of 10% glycerol, 20 mM HEPES, 400 mM NaCl, 1 mM EDTA and Complete Mini-EDTA-free protease inhibitor cocktail. The mixture was incubated on ice for 1 h. The supernatant was collected after centrifugation for 5 min at 15,000×g, and saved as nuclear extracts.

### Western Blotting Analysis

Protein concentrations were determined with the BCA Protein Assay. Equal amounts of protein were electrophoresed on 10% polyacrylamide SDS gel. Then, proteins were transferred onto nitrocellulose membranes. After being blocked for 1 h with 5% skimmed milk in tris-buffered saline containing 0.1% Tween 20, proteins were incubated with the primary antibodies (anti-c-kit antibody, anti-β-MHC antibody, anti-TNI antibody, anti-GATA-4 antibody, anti-caspase-3 antibody, anti-β-actin antibody, anti-RB antibody, anti-pRB antibody, anti-cyclin D1 antibody, 1∶1000 dilution, all from Santa Cruz Biotechnology, Inc USA) and secondary antibodies (anti-rabbit IgG 1∶5000 dilution; Li-cor, USA), the protein bands were detected with the infrared Odyssey imaging System (Li-Cor, USA).

### Statistical Analysis

Values are displayed as mean plus or minus SEM. Comparisons between groups were analyzed by the Student’s t test (two-tailed). Results were considered statistically significant for P values less than 0.05.

## Results

### Isolation, Culture, and Identification of c-kit+ CSCs

**Figure 1:**
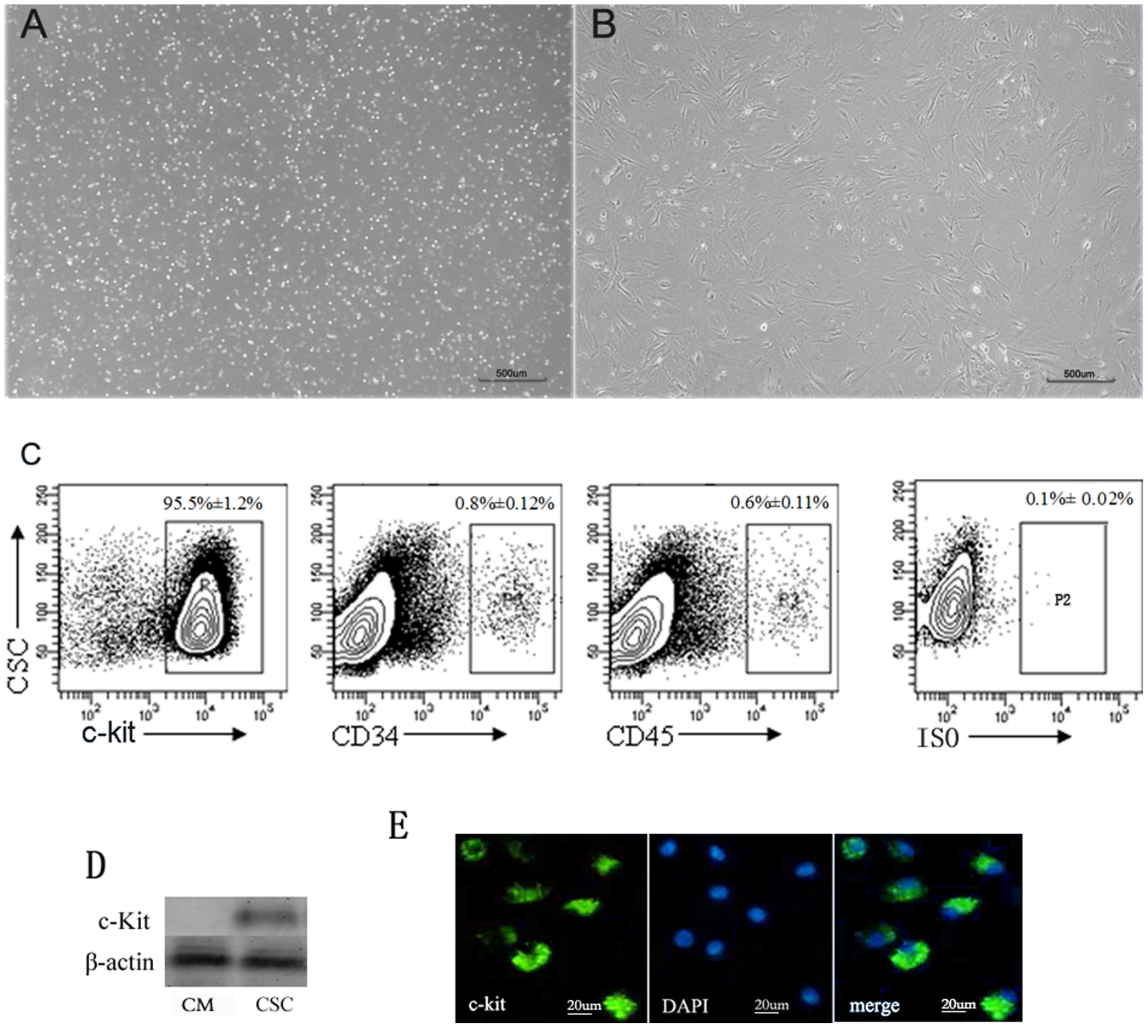
Isolation and identification of c-kit^+^ CSCs. (A) The c-kit^+^ CSCs isolated by immunomagnetic microbeads were almost small, round, phase-bright on the first day. (B) Three days later, c-kit^+^ CSCs gradually attached to the plate, proliferated, and clustered. (C) Quantitative analysis of surface markers of cells expanded in culture by FACS. (D) The expression of c-kit was examined with western blotting in the isolated cells (CSC) and cardiac myocytes (CM). (E) Immunostaining of isolated cells. All experiments were repeated 5 times.

The myocardial tissues from adult mice were subject to digestion to collect total cells. Then, c-kit^+^ CSCs were isolated by immunomagnetic microbeads from the total cells. Under light microscope, the obtained c-kit^+^ CSCs were small, round, phase-bright, and suspended in the medium (Fig. 1A). Three days later, the bright spherical c-kit^+^ CSCs gradually attached to the plate, proliferated, and clustered (Fig. 1B). The expressions of c-kit, CD34, or CD45 were detected by flow cytometric analysis (Fig. 1C). The c-kit^+^ CSCs isolated by immunomagnetic microbeads were with a purity of 95.5%±1.2%.

To further demonstrate that the isolated CSCs were c-kit^+^ CSCs,we performed western blotting and immunostaining. Our research showed that c-kit protein expression is positive in the isolated cells, but not in cardiac myocytes (as a control) (Fig. 1D). Immunostaining also exhibited that the isolated cells are positive in c-kit staining (Fig. 1E).

### Intracellular Iron Content

**Figure 2:**
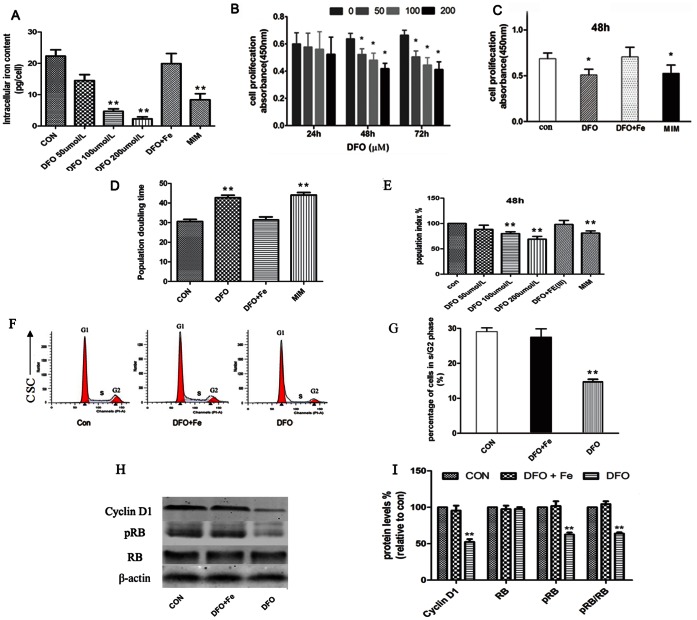
Effect of iron deficiency on cell proliferation and cell cycle. (A) Intracellular iron content was assessed by the colorimetric ferrozine-based assay. DFO and MIM significantly decreased intracellular iron level. (B) Cell proliferation was measured with CCK-8-kit. Result was expressed as the mean of absorbance (450nm) ± SEM. Compared with control group (cells treated with culture medium alone), DFO significantly suppressed c-kit^+^ CSCs proliferation after 48 h or 72h incubation. The inhibitory effect was dose-dependent. (C) DFO and MIM suppressed proliferation of c-kit^+^ CSCs. Fe(III) reversed the suppression effect of DFO on proliferation. (D) Population doubling time was analyzed. Compared with control group, DFO and MIM significantly prolonged the population doubling time. (E) Cell proliferation was further assayed by BrdU incorporation analysis. Cells treated with culture medium alone were defined as con group. (F, G) Cell cycle was analyzed. The c-kit^+^ CSCs were treated with/without DFO (100 µM) or the complex of DFO (100 µM) and Fe(III) (0.5 mM) for 48h. Compared with control group (cells treated with culture medium alone), the proportion of cells in S/G2 phase decreased after DFO treatment for 48 h. (H, I) Effect of iron deficiency on cell cycle-related proteins. The expression of cyclin D1 and the phosphorylation of RB significantly decreased in DFO group. Iron reversed this suppressive effect of DFO. * p<0.05, ** p<0.01 (compared with control group). All experiments were repeated 5 times.

Cells without stimulation were defined as control group. Then, c-kit^+^ CSCs were treated with DFO, MIM, or a complex of DFO and Fe(III). Intracellular iron content was assayed. As shown in Fig. 2A, DFO reduced intracellular iron content in a dose-dependent manner. MIM also obviously decreased the intracellular iron level. When 0.5 mM Fe(III) was added, no significant difference of iron content was seen between DFO+Fe(III) group and control group.

### Effect of Iron Deficiency on c-kit+ CSCs Proliferation

The cell proliferation was determined by CCK-8 colorimetric assay. Cells were stimulated with iron chelators (DFO or MIM) or the complex of DFO and Fe(III). As shown in Fig. 2B, DFO significantly suppressed c-kit^+^ CSCs proliferation. The inhibitory effect was dose-dependent and time-dependent. MIM suppressed proliferation of c-kit^+^ CSCs too. Fe(III) (0.5 mM) reversed the inhibitory effect of DFO on proliferation (Fig. 2C). We also performed the population doubling time analysis and BrdU incorporation assay. As shown in Fig. 2D and 2E, iron chelators significantly increased the population doubling time and reduced the BrdU incorporation of c-Kit^+^ CSCs. Fe(III) reduced this modulation effect of DFO.

### Effect of Iron Deficiency on Cell Cycle

After c-kit^+^ CSCs were incubated with DFO, MIM, or a complex of DFO with Fe(III) for 48 h, cell cycle was analyzed by flow cytometry. In cells treated with DFO, the proportion of S/G2 phase cells significantly decreased. Fe(III) reversed this effect of DFO (Fig. 2F and 2G). To understand the mechanism by which iron deficiency induces the block in G1 phase, we analyzed the expression and phosphorylation of cell cycle-related proteins. Our results exhibited that DFO suppressed the expression of cyclin D1 and the phophorylation of RB. Iron reversed this effect of DFO (Fig. 2H and 2I).

### Effect of Iron Deficiency on the Apoptosis of c-kit+ CSCs

**Figure 3:**
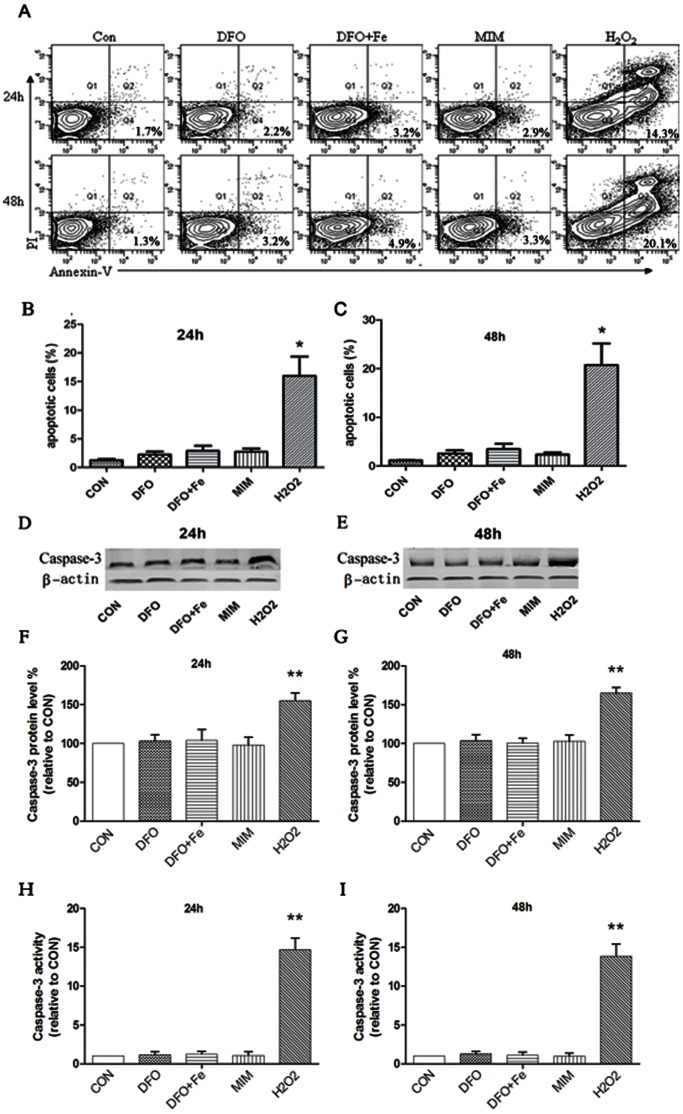
Effect of iron deficiency on c-kit^+^ CSCs apoptosis. (A, B, C) Cellular apoptosis was analyzed. Cells were treated with DFO, MIM, H_2_O_2_, or the complex of DFO and Fe(III) for 24 h or 48 h. H_2_O_2_ was used as positive control. DFO, MIM, and the complex of DFO and Fe(III) didn’t induce cellular apoptosis. Then, caspase-3 expression (D, E, F, G) and activity (H, I) were explored. DFO, MIM, and the complex of DFO and Fe(III) didn’t regulate caspase-3 expression and activity. * p<0.05, ** p<0.01 (compared with control group). All experiments were repeated 5 times.

To determine whether iron deficiency induces the apoptosis of c-kit^+^ CSCs, cells were exposed to DFO, MIM, or the complex of DFO and Fe(III) for 24 h or 48 h. H_2_O_2_ was used to stimulate cells as positive control. The apoptotic cells were quantified by flow cytometry. AS shown in Fig. 3A–3C, neither DFO nor MIM modulated the apoptosis of c-kit^+^ CSCs.

Then, the protein expression and activity of caspase-3 were analyzed. Our results showed that iron chelation didn’t regulate caspase-3 expression and activity (Fig. 3D–3I).

### Effect of Iron Deficiency on c-kit+ CSCs Differentiation

**Figure 4:**
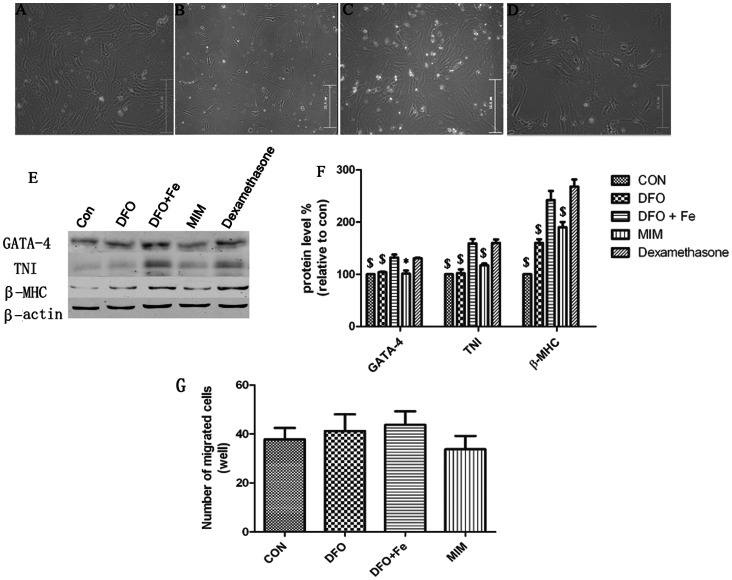
Effect of iron deficiency on c-kit^+^ CSCs differentiation (A–F) and migration (G). Dexamethasone (10^−8^ M) was used to induce differentiation. Cellular morphology and cardiac-specific proteins were assayed. Cells were treated with dexamethasone (A), dexamethasone+DFO (B), dexamethasone+DFO+Fe (C), or dexamethasone+MIM (D). No obvious change in cellular morphology was observed in these groups. The expression levels of cardiac-specific proteins (β-MHC, TNI, and GATA-4) were down-regulated under iron deficiency condition (E, F). (G) Cell migration was assessed. Cells treated with culture medium alone were defined as control group. After c-kit^+^ CSCs were pre-incubated with DFO, MIM, or the complex of DFO and Fe(III) for 24 h, migrated cells were measured by quantitative analysis. Compared with control, c-kit^+^ CSCs migration was not affected by DFO, MIM, or the complex of DFO and Fe(III) (p>0.05). * p<0.05, $ p<0.01 (compared with cells treated with Dexamethasone alone). All experiments were repeated 5 times.

We examined whether iron deficiency affects the differentiation ability of c-kit^+^ CSCs. After cultured in differentiation medium with/without DFO, MIM, or the complex of DFO and Fe(III) for two weeks, the cellular morphology and the expression of cardiac-specific proteins (β-MHC, TNI, and GATA-4) were analyzed. Although no obvious change in cellular morphology was observed in different groups (Fig. 4A–4D), DFO and MIM significantly decreased the expression of cardiac-specific proteins. Fe(III) reduced the inhibitory effect of DFO on cardiac-specific proteins expression (Fig. 4E and 4F).

### Effect of Iron Deficiency on c-kit+ CSCs Migration

To examine whether iron deficiency induces c-kit^+^ CSCs migration, cells were pre-incubated with DFO, MIM or the complex of DFO and Fe(III) for 24 h. Then, transwell migration assays were performed. As shown in Fig. 4G, no significant variation was observed between each group.

## Discussion

The effect of iron deficiency on c-kit^+^ CSCs function was investigated in this study. This paper reported for the first time that iron deficiency inhibits c-kit^+^ CSCs proliferation and differentiation in vitro, but it doesn’t influence c-kit^+^ CSCs migration and apoptosis.

In this study, DFO was used to produce iron deficiency. DFO causes intracellular iron deficiency in vitro by diffusing into cells, where it binds predominantly to the labile iron pool [14]. DFO per se may modulate inflammation status and mimic hypoxia [15]. To confirm that the DFO suppression action in this study is due to iron deficiency, intracellular iron content was assayed. Our results showed that DFO (100, 200 µM) significantly reduced intracellular iron level. When cells were co-treated with Fe(III) and DFO, Fe(III) reversed the down-regulation effects of DFO on c-kit^+^ CSCs function and intracellular iron content. In addition, we found that MIM, an iron chelator structurally distinct from DFO, mimicked the reduction effect of DFO. From these results, we concluded that DFO suppression activity is due to iron sequestration.

Heart failure, a chronic cardiovascular disease, has high morbidity and mortality [16]. More than 70% of CHF patients are accompanied by iron deficiency [17]. There are many causes of iron deficiency in CHF [8], including reduced iron intake due to anorexia, or gastrointestinal blood loss caused by gastrointestinal bleeding from diaphragmatic hernias, ulcers, gastritis, tumors, platelet inhibitors, and anticoagulants. It has also been found that proton pump inhibitors such as omeprazole, which are widely used, also reduce iron absorption. In addition, CHF can cause intestinal cell dysfunction with reduced iron absorption because of bowel edema and other factors. Furthermore, EPO and elevated cytokines (tumor necrosis factor-α, interleukin-6) can also cause abnormalities in iron metabolism. Recent studies have demonstrated that iron plays an important role in CHF progression and prognosis. Iron deficiency with and without anemia is accompanied by reduced aerobic performance and subjective complaints of poor physical condition [9]. This worsening effect is not only directly related to impaired erythropoiesis, but also to marked impairment of oxidative metabolism, cellular energetics, and cellular immune mechanisms [9]. Van et al [18] illustrated that iron regulates the uptake, transport, and storage of oxygen to maintain cells metabolism and cardiomyocytes exercise. Naito et al [19] found that iron deficiency can cause cardiac fibrosis, reduction in erythropoietin levels via STAT3 pathway.

CSCs (c-kit, Sca-1, MDR1, and Isl-1) were multipotent stem cells. Studies have demonstrated that CSCs can proliferate and differentiate into different cells in both vitro and vivo [1], [20], [21]. Dawn et al illustrated that CSCs can differentiate into cardiomyocytes and repair the injured heart [22]. Because c-kit^+^ CSCs are the most potential CSCs, recent studies have focused on the way to regulate its function [23], [24]. However, the specific effect of iron deficiency on CSCs is still not clear. Therefore, we detected the effect of iron deficiency on c-Kit^+^ CSCs migration, proliferation, apoptosis, and differentiation.

First, the effect of iron deficiency on c-kit^+^ CSCs proliferation was investigated. We found that DFO and MIM inhibited the proliferation of c-kit^+^ CSCs in a dose-dependent and time-dependent manner. This inhibitory effect could be reduced by iron. We also observed that DFO and MIM obviously increased the population doubling time and reduced the BrdU incorporation. Thus, we concluded that iron deficiency has an inhibitory effect on c-kit^+^ CSCs proliferation. This finding agrees with previous studies. Gharagozloo demonstrated that iron deficiency inhibits growth and proliferation of Jurkat cells by inhibiting the activity of ribonucleotide reductase and DNA synthesis [13]. Dayani proved that desferoxamine (DFO) inhibits brain cancer cells proliferation [25].

To understand how iron deficiency suppresses cell proliferation, we analyzed the cell cycle of c-kit^+^ CSCs. Some studies have proved that iron deficiency blocks cell cycle in G1 phase in other cells [10], [26]–[28]. Many cell cycle-related factors, such as cyclin D1, GADDA45-α, CDKs, are involved in this block activity [26]–[30]. Among these factors, cyclin D1 is the most classic one. Cyclin D1 enhances the phosphorylation of the RB, which results in transcription of various genes like cyclin E, cyclin A, DNA polymerase, thymidine kinase, and pushes the cell from G1 to S phase [26]–[28]. Our results are consistent with these reports. We found that DFO blocked c-kit^+^ CSCs in G1 phase. The expression of cyclin D1 and the phosphorylation of its downstream target RB were down-regulated by DFO. Iron reversed this suppressive effect of DFO. Taken these results, we concluded that the iron deficiency blocks the cell cycle of c-Kit+ CSC in G1 phase via modulating cyclin D1.

Then, we explored whether iron deficiency modulates cellular apoptosis. In former studies, iron deficiency has been reported to increase cellular apoptosis. Pan demonstrated that iron deficiency induces Jurkat T-lymphocytes shrinkage, membrane blebbing, chromatin condensation and fragmentation, and formation of apoptotic bodies [31]. Our research failed to confirm this pro-apoptotic effect. Although we observed an increasing trend, iron chelators didn’t induce significant cell apoptosis after 24 h or 48 h stimulation. Iron depletion didn’t regulate caspase-3 expression and activity either. Those results demonstrated that iron deficiency doesn’t enhance apoptosis. The difference between our research and the Pan’s may be due to using different cells. We took mouse c-kit^+^ CSCs, while they utilized Jurkat T-lymphocytes.

CSCs migration to injured myocardium is an important process in cardiac function repair. Normally, CSCs reside in cardiac stem cell niches [32]. Under Ischemia/reperfusion injury, CSCs migrate to the injured myocardium [33]. As far as we know, there are no reports of stem cells, but some papers about tumor cells [34], [35], that have investigated the role of iron in cell migration. We found neither DFO nor MIM modulated the migration of c-kit^+^ CSCs. This result indicated that iron deficiency doesn’t affect c-kit^+^ CSCs migration.

CSCs can differentiate into cardiomyocytes [1]. In the present study, we examined whether iron-deficient influences the differentiation of c-kit^+^ CSCs into cardiomyocytes. Cellular morphology and cardiac-specific protein levels were analyzed. Although no obvious changes in the cellular morphology were observed between different groups, a significant difference in the expression levels of cardiac-specific protein was found. Without stimulation, c-Kit^+^ CSCs express a little amount of GATA-4 (an early markers of cardiac lineage), but no β-MHC and TNI (two late markers of cardiac lineage). When cells were treated with differentiation medium, the expression levels of both early and late cardiac-specific markers were up-regulated. DFO and MIM significantly reduced the up-regulation effect of differentiation medium. Fe(III) reversed the inhibition action of DFO. Taken these results together, we demonstrated that iron deficiency modulates the differentiation ability of c-kit^+^ CSCs. This finding is in line with JIa’s research, who revealed that iron may enhance the differentiation of cells in vitro [36].

By analyzing intracellular iron content, we demonstrated that when intracellular iron level is lower than 4.1pg/cell, the proliferation and differentiation ability of c-Kit^+^ CSCs significantly decreases. Since c-Kit^+^ CSCs play an important role in cardiac function repair, this finding helps to explain, at least in part, why CHF patients with iron deficiency have worse prognosis. However, extent to which low-level of serum iron could lead to intracellular iron content lower than 4.1 pg/cell needs to be further explored. We didn’t explore that deep in this study.

In conclusion, iron deficiency modulates c-kit^+^ CSCs functions. It inhibits the cell proliferation ability and reduces the differentiation into cardiomyocyte. Our study provides a possible mechanism of the association between iron deficiency and the poor prognosis of CHF.
